# POSITIVE AGEING AND DEATH OR DYING: A SCOPING REVIEW

**DOI:** 10.1093/geroni/igac059.3132

**Published:** 2022-12-20

**Authors:** Robin Otto, Noelle Fields, Keith Anderson, Michael Bennett

**Affiliations:** University of Texas at Arlington, Arlington, Texas, United States; The University of Texas at Arlington, Arlington, Texas, United States; University of Mississippi, Oxford, Mississippi, United States; University of Texas at Arlington, Arlington, Texas, United States

## Abstract

Ageing world populations and increases in life expectancy have facilitated an interest in developing ageing models that promote inclusivity and positive perceptions of ageing. This scoping review examines how and to what extent research that utilizes successful, active, productive, and healthy ageing framework(s) include death or dying. The study followed Joanna Briggs Institute’s methodological standard for scoping reviews and PRISMA guidelines and conformed to Arskey and O’Malley’s five-stage framework. A thematic analysis was used to identify how research utilizes the concepts of death and dying in the context of positive ageing models. The analysis identified five core themes: (a) the critique that death and dying dimensions in positive ageing models are absent; (b) older adults’ outlooks on death and dying while ageing well; (c) religious and spiritual dimensions of ageing well; (d) negative consequences of positive ageing models without death and dying dimensions; and (e) the future of death and dying in positive ageing models. The results bolster support for a paradigm shift that redefines what it means to age successfully without denying death. Incorporating the topics of death and dying into ageing conversations encourages individuals to ponder their end-of-life preferences and proactively participate in their advanced care plans. Death and dying conversations help care providers support people to live and die in a manner that is meaningful to them and inspire those receiving care to live fully and deeply and to think about the legacy that they want to leave.

